# PD-L2 Is Constitutively Expressed in Normal and Malignant Urothelium

**DOI:** 10.3389/fonc.2021.626748

**Published:** 2021-02-25

**Authors:** Alexander C. Dowell, Haydn Munford, Anshita Goel, Naheema S. Gordon, Nicholas D. James, K. K. Cheng, Maurice P. Zeegers, Douglas G. Ward, Richard T. Bryan

**Affiliations:** ^1^ Institute of Immunology and Immunotherapy, University of Birmingham, Birmingham, United Kingdom; ^2^ Bladder Cancer Research Centre, Institute of Cancer and Genomic Sciences, University of Birmingham, Birmingham, United Kingdom; ^3^ Prostate and Bladder Cancer Research Team, The Institute of Cancer Research, London, United Kingdom; ^4^ School of Health and Population Sciences, University of Birmingham, Birmingham, United Kingdom; ^5^ Department of Complex Genetics and Epidemiology, School of Nutrition and Translational Research in Metabolism, Maastricht University, Maastricht, Netherlands; ^6^ CAPHRI School for Public Health and Primary Care, University of Maastricht, Maastricht, Netherlands

**Keywords:** bladder cancer, immune checkpoint inhibitors, PD-L1 (B7-H1 CD274), PD-L2: programmed cell death ligand 2, normal urothelium

## Abstract

The use of immune checkpoint blockade, in particular PD-1 and PD-L1 inhibitors, is now commonplace in many clinical settings including the treatment of muscle-invasive bladder cancer (MIBC). Notwithstanding, little information exists regarding the expression of the alternative PD-1 ligand, PD-L2 in urothelial bladder cancer (UBC). We therefore set out to characterise the expression of PD-L2 in comparison to PD-L1. Firstly, we assessed PD-L2 expression by immunohistochemistry and found widespread expression of PD-L2 in UBC, albeit with reduced expression in MIBC. We further investigated these findings using RNA-seq data from a cohort of 575 patients demonstrating that PDCD1LG2 (PD-L2) is widely expressed in UBC and correlated with CD274 (PD-L1). However, in contrast to our immunohistochemistry findings, expression was significantly increased in advanced disease. We have also provided detailed evidence of constitutive PD-L2 expression in normal urothelium and propose a mechanism by which PD-L2 is cleaved from the cell surface in MIBC. These data provide a comprehensive assessment of PD-L2 in UBC, showing PD-L2 is abundant in UBC and, importantly, constitutively present in normal urothelium. These data have implications for future development of immune checkpoint blockade, and also the understanding of the function of the immune system in the normal urinary bladder.

## Introduction

Bladder cancer is the 11^th^ most common cancer and the 9^th^ most common cause of cancer mortality in the UK. The majority of cases derive from the urothelium (urothelial bladder cancer - UBC), a specialised epithelial layer lining the urinary tract. The majority of UBC patients present with non-muscle-invasive (NMIBC) disease, whereas the presence or progression to muscle-invasive (MIBC) disease is associated with significantly increased cancer mortality (1).

Immune checkpoint blockade (ICB) has heralded a new era in cancer therapy. UBC is an ideal setting for ICB as evidenced by landmark trials and FDA licencing of anti-PD-1 and anti-PD-L1 antibodies (2). However, whilst 15%–29% of patients exhibit an objective response to checkpoint inhibitors, the majority of patients fail to respond (3). As such, many questions remain to be answered in order to permit informed clinical decision-making, alongside continued therapeutic development and further advancement of the field.

PD-1 and its ligands, PD-L1 and PD-L2 have a prominent role in maintaining self-tolerance under normal physiological conditions, limiting T cell activation and proliferation in peripheral tissues. The majority of investigation has understandably focussed on PD-L1 expression by tumor cells, given the hitherto-proposed restricted expression pattern of PD-L2: in contrast to the widespread expression of PD-L1, PD-L2 expression was believed to be restricted to macrophage and other professional antigen presenting cell types (4). Recently, however, PD-L2 has been identified as expressed by a range of solid tumors including non-small cell lung carcinoma, head and neck squamous cell carcinoma, gastric, oesophageal, renal cell carcinoma, and hepatocellular carcinoma (5–9). Importantly PD-L2 may be an independent predictor of response to PD-1 blockade (8). Very limited knowledge exists for UBC. A previous case report described amplification of *PDCD1LG2* (PD-L2) (10), yet TCGA data indicate that this is a rare event. Published RNA-seq data from 34 UBC samples (of unknown grade and stage) indicated PD-L2 is expressed in addition to PD-L1 (8). While a recent study by Yang et al. indicated increased expression of PD-L2 associated with higher stage (11), a high proportion of (~20%) squamous cell and other variant tumors were investigated and are shown to express higher PD-L2 levels. Given the growing use of ICB in clinic, it is vital to understand the expression of these ligands in target tissues.

We set out to characterise tumoral expression of both PD-1 ligands, PD-L1 and PD-L2, in UBC patients spanning the full range of grades and stages of disease. We show PD-L2 is widely expressed by bladder tumor cells; however, protein expression is inversely associated with disease stage. We go on to show for the first time that PD-L2 is constitutively expressed by normal urothelium, both in healthy individuals and those with UBC. These data highlight a previously unknown role for PD-L2 in the immune privileged site of the urinary bladder, and may further inform the use of PD-1/-L1 immune checkpoint blockade.

## Material and Methods

### Patient Samples

Tissue microarrays (TMAs) of formalin fixed paraffin embedded (FFPE) tissue sections of newly-diagnosed primary UBCs were obtained from the Bladder Cancer Prognosis Programme (BCPP — clinicaltrials.gov identifier NCT00553345, ethics approval 06/MRE04/65) (12). Tissue collection was performed at initial TURBT as previously described (12). Patients were recruited between 2005 and 2011 from nine hospitals in the West Midlands region of the UK and gave informed consent for enrolment into BCPP on the basis of initial cystoscopic findings suggestive of primary UBC. All patients were newly-diagnosed, had not received UBC treatment prior to biospecimen collection, and were subsequently treated and followed-up according to contemporary guidelines (including re-resection where indicated). Inclusion and exclusion criteria are detailed elsewhere (12).

Tumor grade and stage records were amended according to results of re-resection or cystectomy (where performed). We used the 1973 grade classification (from grade 1, well differentiated, to grade 3, poorly differentiated, defined by cellular appearances) as it was in universal use in the UK at the time of patient recruitment, and is also the basis for the EORTC and EAU NMIBC risk tables (13, 14). Tumor stages range from Ta/T1 through to T4, defined by the level of infiltration from the urothelium (Ta), into the submucosa (T1), the underlying detrusor muscle (T2), perivesical fat (T3), and ultimately into surrounding structures (T4). Diagnostic FFPE tumor samples were retrieved from local histopathology departments, and 10% of all such samples underwent expert pathological review as part of routine quality assurance. All included tumors were purely or predominantly transitional cell carcinomas (TCC). Patient demographic information is presented in **Table 1**.

**Table 1:** Patient demographic – TMA immunohistochemistry cohorts.

	Immunohistochemistry (PD-L1)			Immunohistochemistry (PD-L2)		
	n = 123			n = 146		
	G1	G2	G3	G1	G2	G3
pTa	20	22	8	27	29	9
pT1	1	10	29	1	10	34
T2+	0	1	32	0	1	35
Age	84 (48-102) years					
Gender	82.4% Male 11.6% Female					

### Immunohistochemistry

Briefly, FFPE bladder tumor TMAs were de-waxed and rehydrated; after antigen retrieval, slides were blocked with 2% horse serum, then stained with anti-PD-L1 (clone: E1L3N; Cell signalling), anti-PD-L2 (clone: HPA013411; Sigma, UK) or isotype control antibodies overnight at 4°C. Following washing in TBST, slides were treated with HRP secondary reagents (Vector, UK) and developed using TSA reagents (Perkin Elmer) and counterstained with DAPI (Cambridge biosciences, UK).

### Analysis of Staining

Stained sections or TMA slides were pictured using a Vectra multi-fluorescent microscope, and images analysed by inform 2.3 software (Perkin Elmer). Software and images were additionally validated by a trained pathologist. All images are at x20 magnification.

### RNA Extraction and qPCR

RNA was extracted and converted to cDNA as previously described (15). qPCR was performed using Taqman probes (Thermo Fisher Scientific); PD-L1 (Hs_01125269_m1), PD-L2 (Hs_01057777_m1) and GAPDH (NM_002046.3). Samples were run in triplicate including no reverse transcriptase controls on an ABI Prism 7500 Sequence Detection System (Applied Biosystems). Ct value for each sample were determined using SDS v1.7 software (Applied Biosystems) and expressed relative to the GAPDH.

### RNA Sequencing

For our BCPP RNA sequencing (RNAseq), libraries were prepared using TruSeq Stranded Total RNA with Ribo-zero Gold (Illumina) and paired-end sequenced (Illumina HiSeq/NextSeq). After QC, fastq data were aligned to GRCh37 reference annotation and reads counted with STAR aligner (v2.5.2b). Read count data from our cohort was combined with publicly available data from TCGA-BLCA (16) and the Hedegaard et al. cohort (17). Samples were excluded based on histology (to remove variant non-TCC samples) and to exclude samples with low read depth: a) BCPP cohort - 85 RNA-sequencing samples in total, 50 samples retained due to excluding those with low read-depth; b) TCGA cohort - 408 RNA-seq samples, 351 samples retained due to excluding variant histology (no samples excluded due to low read-depth); c) Hedegaard cohort- 460 RNA-seq samples, 206 samples retained due to excluding those with low read-depth. Samples with known tumor stage were then selected, see **Table 2**. Detailed histopathology for the combined RNA-seq cohorts can be found in their accompanying publications (12, 16, 17). To account for the three different cohorts, the analysis commenced from raw read counts. Normalization was performed on the combined dataset using the *voom* method implemented in the *limma* package (v.3.36.2) and batch-effect correction was applied using the *ComBat* method implemented in the *sva* package (v.3.28.0) using R (v3.5.1). RNAseq of BCPP samples was carried out by Genomics Birmingham, Department of Experimental Medicine, University of Birmingham, UK.

**Table 2:** Patient demographic – RNA-seq cohorts.

	BCPP RNA-seq cohort			Combined RNA-seq cohorts
	n = 44			n = 575
	G1	G2	G3	
pTa	15	2	7	170
pT1	0	1	19	66
T2+				339
Age	70 (44–87) years			69 (34–94) years
Gender	88.6% Male 11.4% Female			77.6% Male 22.4% Female

### PD-L2 Elisa

Mid-stream urine samples were collected from individuals without a history of bladder cancer. Samples were spun at 10,000g for 10 min prior to analysis. A PD-L2 Elisa kit (Cat. BMS2215, Thermo Fisher Scientific) was used following the manufactures instructions.

### Statistical Analysis

Data was analysed using GraphPad Prism software version 8.4.2 (GraphPad Software Inc., USA). Statistical tests are as indicated.

## Results

### PD-L2 Protein Expression

**Figure 1:**
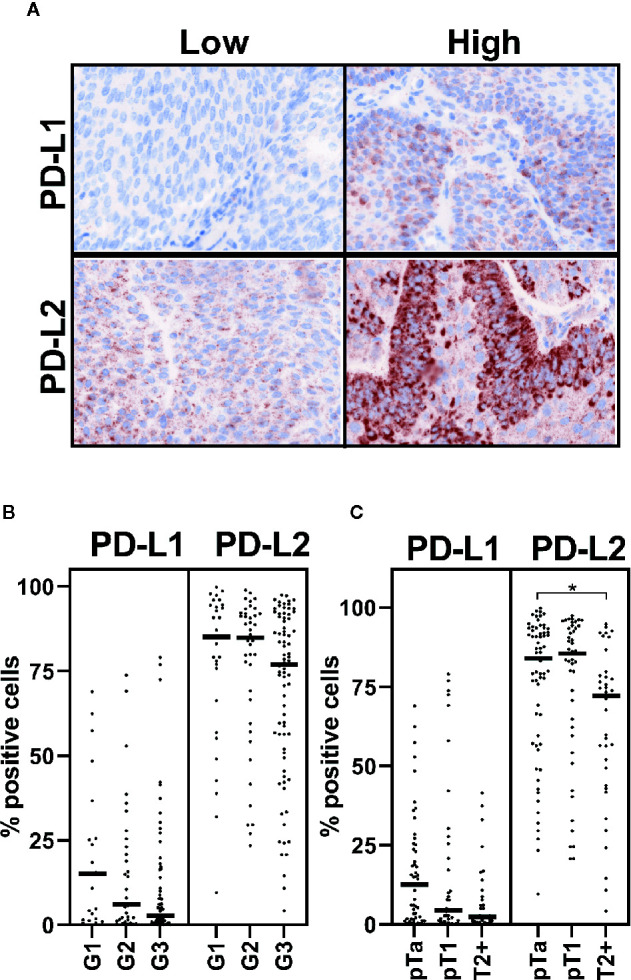
PD-L2 may be altered in muscle-invasive disease. PD-L1 and PD-L2 expression on tumor cells was investigated by immunohistochemistry staining of TMA. Staining was visualised by tyramine signal amplification. Data is presented from 123 and 146 patient samples, which were suitable for analysis, respectively. **(A)** Representative cases with high or low levels of PD-L1 and PD-L2 staining, as indicated. PD-1 ligand (as indicated) staining (brown), with nuclear counter stain (blue). **(B, C)** Positive immunohistochemical staining was assessed by inForm automated tissue analysis, and is presented as percentage positive tumor cells. Expression of PD-1 ligands is shown in respect of grade **(B)** and stage **(C)** One-way ANOVA with Tukey’s multiple comparison test; *p = 0.0296.

To define whether tumor cells express PD-L2 protein, we employed the use of TMAs comprising a range of bladder cancer stages and grades (**Table 1**). These were stained for PD-L1 and -L2 by immunohistochemistry and visualised by tyramine signal amplification. Positive tumor cell staining was assessed and scored using inForm automated tissue analysis. We found abundant widespread expression of PD-L2 on tumor cells in most cases, in contrast to the focal expression of PD-L1 (**Figure 1A**). There was no significant association between PD-L1 expression and grade or stage, whereas there was a significant decrease in PD-L2 expression associated with muscle-invasive disease (pTa vs pT2+, p=0.0296; one-way ANOVA with Tukey’s multiple comparison) (**Figures 1B, C**).

### *PDCD1LG2* (PD-L2) Gene Expression

**Figure 2:**
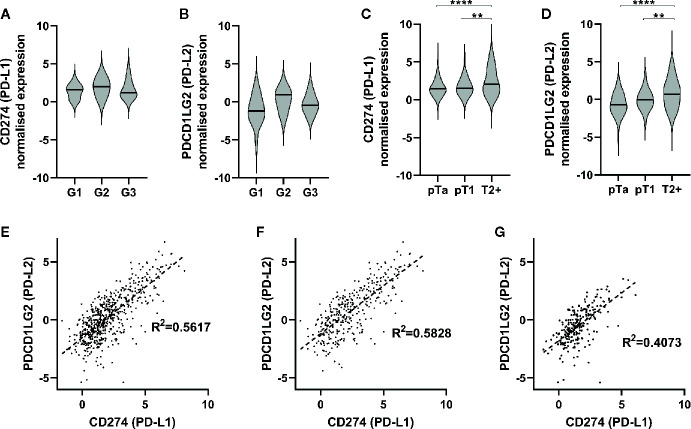
PD-1 ligands are expressed in urothelial bladder cancer (UBC), and are associated with increasing stage. **(A, B)** RNA-seq was performed on tumor samples from 44 non-muscle invasive bladder cancer patients (pTa-pT1) and normalised. *CD274* (PD-L1) **(A)** and *PDCD1LG2* (PD-L2) **(B)** gene expression is presented in respect of tumor grade (G1 n=15, G2 n=3, G3 n=26). **(C, D)** RNA-seq data from two publicly available data sets with tumor stage data were included for further analysis. Data shown for 575 patients (pTa n=170, pT1 n=66, T2+ n=339). CD274 (PD-L1) **(C)** and PDCD1LG2 (PD-L2) **(D)** gene expression is shown with respect to tumor stage. One-way ANOVA with Tukey’s multiple comparison test; **p = < 0.01, ****p = < 0.0001. **(E–G)** Correlation of CD274 and PDCD1LG2 gene expression from combined RNA-seq cohorts. **(E)** All UBC samples (n = 575), **(G)** MIBC (n = 339), **(F)** NMIBC, and (n = 236).

Regarding *PDCD1LG2* (PD-L2) gene expression in UBC, we utilised RNA-seq data from our own analysis of 44 NMIBCs (18). These data confirmed expression of *PDCD1LG2* (PD-L2) in addition to *CD274* (PD-L1), although no effect of tumor grade was evident on expression of either gene in this small cohort (**Figures 2A, B**). Since these RNA-seq data were derived from NMIBCs we combined our data with two public datasets to further investigate expression of PD-L1 and PD-L2 in a combined cohort of 575 NMIBC and MIBC patients. Analysis of expression of *CD274* (PD-L1) (**Figure 2C**) and *PDCD1LG2* (PD-L2) (**Figure 2D**) is shown with respect to stage - there was a significant increase in PD-L1 (*CD274*) expression associated with increased tumor stage (pT1-T2+ p=0.0031, pTa-T2+ p=<0.0001; one-way ANOVA with Tukey’s multiple comparison). There was a similar strong association of PD-L2 (*PDCD1LG2*) with increased stage (pT1-T2+ p=0.0021, pTa-T2+ p=<0.0001). As expression of both genes increased with increased stage, we questioned whether their expression was correlated and demonstrated this to be the case (R² = 0.5617) (**Figure 2E**). This correlation was most evident in MIBC (T2+) patients (R² = 0.5828) compared to NMIBC patients (R² = 0.4073), (**Figures 2F, G**). These data thus indicate that PD-L2 mRNA is expressed at higher levels in more advanced disease, in contrast to the relationship demonstrated for PD-L2 protein expression.

### *PDCD1LG2* (PD-L2) and PD-L2 are Constitutively Expressed By Normal Urothelium

**Figure 3:**
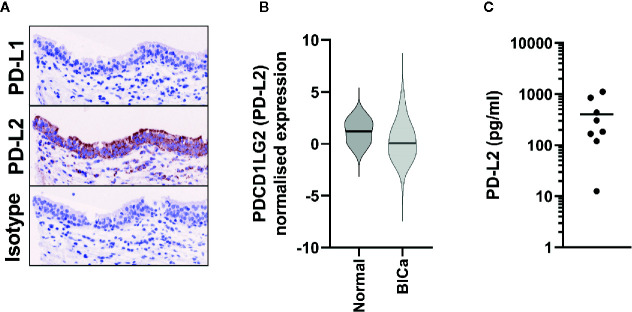
PD-L2 is constitutively expressed in Normal Bladder tissue. **(A)** Representative immunohistochemistry staining of either PD-L1 or PD-L2 on normal ureter urothelium from TMAs. **(B)** *PDCD1LG2* (PD-L2) gene expression from TCGA data from normal bladder tissue (n = 19), in comparison to all bladder cancer samples (n = 575). **(C)** Urinary PD-L2 levels were determined by ELISA in mid-stream urine samples from 8 healthy volunteers.

In individuals diagnosed with benign urothelial lesions we also assessed the expression of PD-L2 by immunohistochemistry and detected PD-L2 expression in all samples tested (**Supplementary Figure 1**). Furthermore, although access to completely normal urothelium is challenging, two cores from normal human ureter were included on each TMA, and the urothelial component stained strongly for PD-L2 (**Figure 3A**).

In the benign urothelial lesions we also detected *PDCD1LG2* expression by qPCR in all samples tested (**Supplementary Figure 1**), and the levels of expression were similar to those found in the NMIBC tumor samples. In support of these findings, *PDCD1LG2* (PD-L2) expression was detected in all 19 normal tissues in the TCGA-BLCA RNA-seq cohort (**Figure 3B**).

As a final orthogonal confirmation of this constitutive expression, we hypothesised that PD-L2 could be shed into the urine from the urothelium. Hence, we assessed its presence in urine samples from eight healthy donors by ELISA; PD-L2 was present in all samples (**Figure 3C**).

These data show that, in contrast to the previous view of PD-L2 being restricted to specific immune cell lineages, PD-L2 is expressed by normal urothelium and benign urothelial lesions, and urothelial carcinomas.

## Discussion

The use of PD-1 and PD-L1 inhibitors is now commonplace in many clinical settings, including the treatment of MIBC, and trials in NMIBC are ongoing (19). Notwithstanding, little information exists regarding the expression of the alternative PD-1 ligand, PD-L2. In this study, we have demonstrated by orthogonal approaches that PD-L2 protein is constitutively expressed by both benign and malignant urothelium.

The data presented by Yang et al (11). appear at odds with the data presented here. However, a number of confounding factors exist; as mentioned, the Yang et al. study comprises a high proportion (~20%) of variant tumors, which are shown to express high PD-L2 levels, whereas our study uses only transitional cell carcinomas. Furthermore, Yang et al. demonstrate a marked variation in the levels of expression across tumor stages [T1; 12/35 (34%), T2; 16/32 (50%), T3 4/16 (25%), T4; 8/9 (89%)]. The data presented by Yang et al, however, are in keeping with the RNAseq data that we present, indicating increased expression with increased stage, in contrast to our own IHC data. A notable difference between our IHC method and Yang et al. is their use of a polyclonal antibody, binding multiple epitopes across PD-L2, whereas we used a monoclonal antibody binding a single epitope. Unlike PD-L1, PD-L2 is highly sensitive to protease-mediated cleavage (20). Using the IProt-Sub algorithm (21) to determine cleavage sites reveals multiple cleavage sites between the transmembrane domain and the antibody binding site for our monoclonal antibody (22). Importantly, this includes cleavage sites for the metalloproteases MMP-2, -3, -12, and -13, with several MMPs elevated in bladder cancer and associated with increased stage (23). Therefore, our data indicate that it is highly likely that PD-L2 is cleaved from the cell surface in more advanced stages of UBC. This highlights an important biological modification that would likely influence PD-L2 function. Such phenomena are well recognised in UBC and other malignancies (24). Further proteomic studies will be required to understand the cleavage and subsequent shedding of PD-L2 from the cell surface in advanced UBC.

PD-L2 was originally identified at mRNA level in murine antigen-presenting cells, and was also documented in some normal tissues; however, neither murine nor human urinary bladder were studied (25). Expression at the protein level, however, did not correlate with the wider mRNA expression pattern in the mouse (26, 27). These early data highlighted that, unlike PD-L1 which is widely inducible on many cell types by inflammatory cytokines, expression of PD-L2 is restricted. High gene expression did not correlate with expression of protein in these earlier studies, and these data are in-keeping with our observation of increased mRNA expression, but reduced or lost protein expression, in advanced disease. These data highlight that caution should be used when studying PD-L2 gene expression data; likewise, to fully contextualise IHC data, it may also be helpful to have accompanying RNA data.

An important question exists as to the role of PD-L2 in normal physiology. PD-L2 binds to PD-1 with higher affinity than PD-L1, inducing qualitatively and quantitatively different signalling. Expression of PD-L1 is thought to be *via* a mechanism which exists to restrict tissue damage during ongoing inflammatory responses. As PD-L1 is induced by inflammatory cues, this may suggest differing roles. Indeed, the evolutionary divergence of PD-L1 and PD-L2 has been traced to placental mammals, suggesting a critical role in maintaining tolerance out with the context of inflammation (28). The urinary bladder is regarded an immune-privileged site, which is exposed to a diverse array of chemicals, toxins and potential antigens; there is, therefore, a requirement for the maintenance of the integrity of the urothelium and consequently tolerance. The constitutive expression of PD-L2 in the human bladder thus suggests a role in maintaining tolerance.

Soluble PD-L2 (sPD-L2) in plasma has been described in a number of studies. Interestingly, in a number of tumor settings, sPD-L2 is reduced in comparison to heathy controls (29–31). The role and functional consequence of sPD-L2 thus remains unclear and is beyond the scope of this study. Here, we have shown sPD-L2 in urine; as PD-L2 is highly sensitive to protease digestion, whether sPD-L2 in this context is functional is unknown. Likewise, whether sPD-L2 may cross the urothelium rather than being excreted is also unclear. As such the consequence of sPD-L2 requires further investigation.

Interestingly, two recent case reports highlighted non-bacterial cystitis as a potential side effect of PD-1 blockade in bladder cancer patients (32), consistent with reports of non-bacterial cystitis adverse events from other trial data. It is interesting to note that PD-1 knockout is required, but not sufficient alone, to induce a murine model of autoimmune cystitis (33). Redundancy within control of tolerance would be in-keeping with the low rate of bladder-related adverse events in trials of PD-1 blockade. Although rare, these events may provide evidence of the role of PD-L2 in maintenance of tolerance in the urinary bladder.

It should be noted several limitations exist. Firstly, RNAseq data and protein data are derived from different cohorts, and as such are not directly comparable, although both cohorts are relatively large datasets of predominantly or pure TCC, allowing a general comparison. Ideally further investigation of matched samples would be performed in future studies. Secondly, the use of tissue microarray allowed many samples to be tested while limiting technical variation. However, PD-L1 is known to be focal, generally around areas of inflammation such as the tumor margin. Due to the smaller areas used in TMAs, there is an increased risk of overlooking areas of high PD-L1 expression, which may underestimate the expression of PD-L1. However, given the widespread expression of PD-L2 there should not be a significant effect on these data. Lastly, although the TMAs were constructed to represent patients recruited at each hospital site, the TMAs used here contained a lower proportion of female patients than would be expected.

PD-1 blockade inhibits binding of PD-L1 and PD-L2, whereas PD-L1 blockade alone renders PD-L2 signalling intact. Both PD-1 and PD-L1 blockade are licenced for the treatment of MIBC (2), and trials in NMIBC are ongoing (19). Our data have implications for future developments in ICB for UBC, and indicate that greater understanding of PD-L2 signalling during PD-1/L1 blockade is required.

## Data Availability Statement

The datasets presented in this study can be found in online repositories. The names of the repository/repositories and accession number(s) can be found below: The RNA sequencing dataset from the BCPP cohort is available online in the EGA repository under the accession EGAS00001004358.

## Ethics Statement

The studies involving human participants were reviewed and approved by East Midlands Health Research Authority. The patients/participants provided their written informed consent to participate in this study.

## Author Contributions

AD, AG, DW, and RB devised the study and methods used. AD, DW, and RB provided funding for this project. NG, NJ, KC, MZ, DW, and RB were responsible for conceptualization, funding, and sample contribution to and from BCPP. AD, HM, AG, NG, DW, and RB carried out experiments and performed formal analysis. All authors were involved in the writing and review of the manuscript. All authors contributed to the article and approved the submitted version.

## Funding

The BCPP study was funded by Cancer Research UK (C1343/A5738), the University of Birmingham and the Birmingham and The Black Country and West Midlands North and South Comprehensive Local Research Networks. BCPP is under the sponsorship of the University of Birmingham.

## Conflict of Interest

RB has contributed to advisory boards for Olympus Medical Systems and Janssen, and undertakes research funded by UroGen Pharma, QED Therapeutics and Janssen. NJ has contributed to advisory boards for Merck USA and Pierre Fabre.

The remaining authors declare that the research was conducted in the absence of any commercial or financial relationships that could be construed as a potential conflict of interest.

## Supplementary Material

Supplementary Figure 1 PD-L2 Gene and protein expression in benign bladder conditions. **(A)** Samples from two biopsy samples found to be benign were tested by qPCR for *PDCD1LG2* expression; shown for comparison are results from G3 pT1 patient samples. **(B)** A representative example of immunohistochemistry staining of benign bladder tissue samples included in TMA, PD-L2 expression (brown), with nuclear counter stain (blue).
