# Supplementary figures and images for: PD-L2 Is Constitutively Expressed in Normal and Malignant Urothelium
Source: Front Oncol. 2021 Feb 25;11:626748. doi: 10.3389/fonc.2021.626748 (PMC7951139; doi:10.3389/fonc.2021.626748)

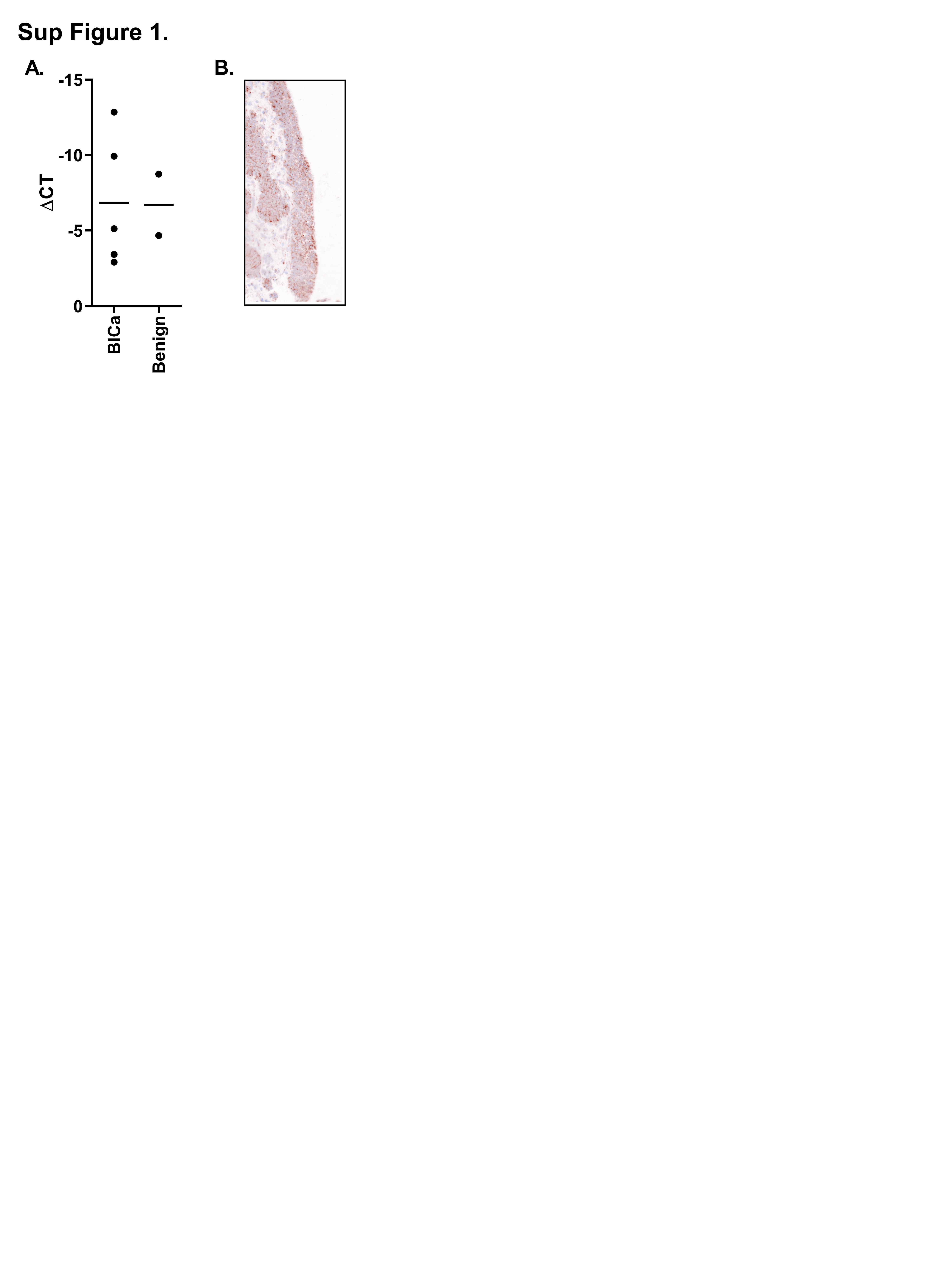

Supplement: Supplementary Figure 1 — PD-L2 Gene and protein expression in benign bladder conditions. (A) Samples from two biopsy samples found to be benign were tested by qPCR for PDCD1LG2 expression; shown for comparison are results from G3 pT1 patient samples. (B) A representative example of immunohistochemistry staining of benign bladder tissue samples included in TMA, PD-L2 expression (brown), with nuclear counter stain (blue). [file Image_1.jpeg]
